# Department of Therapeutics

**Published:** 1894-06

**Authors:** 

**Affiliations:** Demonstrator of Physiology and Lecturer on the History of Medicine in the Medical Department of the University of Texas, etc.; Galveston


### Current Medical Literature.

### DEPARTMENT OF THERAPEUTICS.

EDITED BY DAVID CERNA, M. D., PH. D.,
Demonstrator of Physiology and Lecturer on the History of Medicine in the Medical Department of the University of Texas, etc.

THE THERAPEUTIC USES OF OXYGEN GAS.—Summing up his conclusions regarding the uses of oxygen gas by inhalation in medical treatment, G. Newton Pitt (*Medical Press and Circular*, January 24, 1894), says: 1. The inhalation of oxygen is of marvelous value in some cases of severe pneumonia, especially when there is much lividity and cardiac failure and at the crisis. It fails in other cases, and I should be inclined to suggest that where the condition is one of mainly cardiac failure and collapse, more benefit is obtained than in cases where the serious condition is especially due to a widespread œdema or bronchitis; but on this difference between the two classes of cases I should be glad to learn the experience of others. Some cases of severe bronchitis and asthma have, however, been benefited by oxygen. In one case of acute upon chronic bronchitis in an elderly lady, I saw some, though not very marked, relief of the dyspnœa. 2. In cases of empyema, pneumothorax, and pleuritic effusion, great relief can be afforded to the dyspnœa and cardiac failure until operative measures are undertaken. 3. Cases of feeble patients with phthisis may be relieved, but more often there is no marked change. 4. Cases of weakly convalescents and feeble cardiac cases will often derive great benefit, and inhalations may be given periodically for weeks; but oxygen will not restore to health; it must simply be used as an adjuvant. 5. Cases of chlorosis, pernicious anæmia, and leucocythæmia receive great temporary benefit, but the oxygen must be supplemented by other drugs. 6.

Conditions of asphyxia and lividity from respiratory engorgement due to cerebral failure and also coma from various causes may be relieved. 7. Its value in uræmia, though insisted upon by French writers, is still problematical. 8. It may also be of value in diminishing the risks of anæsthesia.

---

THE ANTIPYRETIC ACTION OF GUAIACOL LOCALLY APPLIED.—Julius Friedenwald and H. H. Hayden, of Baltimore, (*New York Medical Journal*, April 14, 1894), published an excellent clinical report of the antipyretic action of guaiacol locally applied. Seventeen cases were treated, comprising 2 of pneumonia, 2 of typhoid fever, 2 of pulmonary tuberculosis, 1 of malaria, 2 of influenza, 1 of acute articular rheumatism, and 1 of erysipelas, in all of which the results were satisfactory. The local use of the remedy was in every case accompanied with profuse diaphoresis, and in some with marked general depression, but no symptoms of poisoning were observed. The authors, from the results obtained, conclude: 1. That this drug has a powerful antipyretic action, occasioning a reduction of from one to four degrees of temperature in from one to four hours. 2. That in all cases this reduction of temperature is accompanied by profuse diaphoresis, which may or may not be accompanied by a chill or chilly sensation. 3. That great exhaustion is frequently produced. 4. That the effects may be obtained from comparatively small doses (from thirty to fifty drops), and that great care should therefore be exercised in the use of the drug. The drug should be applied but once or twice daily, and the initial dose should not be above thirty drops. 5. That the effect produced by guaiacol, though more powerful, is the same as is obtained from most of the other antipyretics of the coal-tar series, and that the same care must therefore be exercised as with the other preparations. Its effect differs from the *stimulating* cold bath in being *depressant*. 6. That the main indication for its use is in diseases accompanied by high fever in which the cold bath can not be applied. It may therefore, be especially useful in typhoid fever, as well as in all other diseases accompanied by high fever in which irritability of the stomach prevents the use of other antipyretics.

---

THE HYPODERMATIC USE OF PILOCARPINE IN FACIAL ERYSIPELAS.—Julius L. Salinger, of Philadelphia (*Therapeutic Gazette*, March 15, 1894), give the details of two cases of facial erysipelas, out of twenty-eight cases observed, in which the subcutaneous employment of pilocarpine gave satisfactory results. Summing up these results, the author says that: In all the cases treated by this method (twenty-eight) the disease was comparatively severe. In none did the treatment last longer than eight days, and quite a number recovered in four days. Albuminuria, to a greater or less extent, was present in twenty-six cases, and lasted throughout the course of the disease. In the severest cases quite appreciable amounts could be obtained by the cold test with nitric acid. In none of the cases were tube-casts present. Four of the patients suffered from retention of urine. The good results obtained by pilocarpine must be ascribed to its action on the skin and subcutaneous tissues. Perhaps the sweating induced by its administration opens a passage for the expulsion of the bacilli which are responsible for this disease. Certain it is that larger quantities of urine are passed and retention rapidly relieved by pilocarpine. The advantage of administering pilocarpine hypodermically is to be found in its rapid action. In order to obtain the best results, the full physiological action of pilocarpine must be produced; that is to say, that unless marked sweating, increased salivation, and increased diuresis are noticed, good results will be looked for in vain. The only contraindications to its use would seem to be in cases of actual organic disease of the heart. Where cardiac disease is present, pilocarpine may have entirely too depressing an effect upon the circulation, nor would it be a safe remedy for old, enfeebled, and cachectic persons. When erysipelas occurs as a complication in another disease, pilocarpine has not shown itself to be effectual. It seems that, where erysipelas occurs as a secondary disease, the process is more severe. Hence so called idiopathic erysipelas is the only form of the disease in which pilocarpine may be safely and advantageously administered.

---

PARAFORM—AN ANTISEPTIC.—At a recent meeting of the Berlin Medical Society, Aronson (*Medical Week*, 1894, Vol. II, p. 143, *American Medico-Surgical Bulletin*, May 1, 1894,) recommended paraform (polymeric formic aldehyde) as an eligible antiseptic and disinfectant, particularly serviceable for disinfection of the intestinal tract. In surgery it may be employed for the disinfection of everything used for dressing, as well as of the operating room. Its chief advantage is said to consist in its retaining its activity while in a vaporous state. It is described as a white, crystalline substance, insoluble in water. On comparing the effects of paraform with those of iodoform, dermatol, salol, betanaphthol, and benzonaphthol, the author found that only paraform and betanaphthol prevented the development of bacteria. To arrest the growth of the typhoid bacillus, it was necessary to employ a 1:3000 solution of betanaphthol; while a 1:50,000 solution of paraform sufficed to do the same. With 5 centigrammes (¾ gr.) of paraform, 200 grammes (6½ fl. oz.) of urine were sterilized; whilst 15 centigrammes (2¼ gr.) of betanaphthol were required for the same purpose. The author has taken as much as 5 grammes (77 gr.) of paraform, without experiencing the slightest untoward effect. The physiological action of paraform is stated to resemble that of calomel, doses exceeding 3 or 4 grammes cause copious stools, while smaller doses rather constipate. Dr. A. has treated about twenty children affected with cholera nostras, by administering paraform in doses of 0.5-1 gramme (7½-15 gr.), obtaining the same effects as from the use of calomel. Pure paraform is said to be irritating to wounds; and when introduced into the organism, this remedy is believed to be volatilized in part, for, on being exposed for some thirty-six hours to a temperature of 38° C. (100.4 F.)—about the body temperature—it loses 10 per cent. of its weight.

---

THE MEDICINAL TREATMENT OF TYPHLITIS.—Grasset (*Rev. Gen. de Clin. et de Therap. University Medical Magazine*, May, 1894,) summarises his views on the treatment of this affection. The indications vary according as the attack is acute and inflammatory, or one of recurrent typhlitis between the attacks, or typhlitis with persistent fecal engorgement. In the last variety medicinal treatment is rarely available, and recourse must be had to operative interference. In recent typhlitis with acute exacerbations Grasset gives the following directions: 1. A warm bath is to be given lasting from half an hour to an hour. 2. Every hour the patient is to receive a teaspoonful of a mixture consisting of one part each of castor oil and almond oil, and two parts of syrup of lemon, until the bowels move freely. 3. Inunctions of an ointment of mercury and belladonna are to be made over the right iliac region, and are to be followed by the application of a large, thin, hot flaxseed poultice. In obstinate cases a drop of croton oil may be added to the purgative mixture.

In recurrent typhlitis in the intervals between the attacks he advises: 1. A diet which leaves little residue. 2. The application once a week of the actual cautery to the painful and indurated region, also daily frictions of an ointment of belladonna. 3. For the relief of constipation, a pill at bed-time, containing one-sixth of a grain each of powdered belladonna, extract of belladonna, and podophyllin. 4. For the purposes of securing intestinal antisepsis, a powder or capsule, containing seven or eight grains of benzo-naphthol, before and after each meal.

---

QUININE AS A REMEDY FOR ENURESIS.—Under this title, Charles S. Potts, of Philadelphia (*Therapeutic Gazette*, April 16, 1894), publishes an interesting communication, and reports two cases of enuresis, successfully treated with quinine in full doses. The author believes that the results obtained seem to confirm Wood's theory of the choreic movement. In one of the cases reported the drug was given in as high doses as 40 grains a day, with the result that the enuresis was corrected. It was in this very case that the disease returned on the suspension of the quinine, but again disappeared on the re-administration of the medicament, showing a distinct beneficial action of the cinchona alkaloid. The author remarks, in speaking of enuresis. "It would seem that in this condition, as in chorea, quinine may prove useful to produce a rapid effect, but to secure permanent results the system must be built up by tonics and other appropriate measures in order to increase the general muscular tone, which it will be remembered is also lacking. If subsequent trials should give similar good results, the use of quinine should be preferable to that of belladonna, the old stand-by in this class of casses."

---

BISMUTH SUBGALLATE IN DERMATOLOGY.—It will be remembered that the common name of *dermatol* has been given to the subgallate of bismuth; the drug has been highly recommended locally applied in the treatment of skin diseases. In a recent article, J. Abbot Cantrell (*Therapeutic Gazette*, April 16, 1894), of Philadelphia, does not seem to have obtained good results from the use of this medicament. He summarises his results as follows: 1. The drug may not be toxic. I did not investigate this property, as I had occasion to use it externally only. 2. It is not superior to other iodoform substitutes, such as iodol and aristol. 3. It does not hinder discharge; on the contrary, it increases it. 4. It is decidedly irritating. 5. It is a stimulant rather than an astringent; in fact, it acted almost as a caustic in one or two cases. 6. It can not take the place that has been alloted to it. 7. I think we have more promising remedies.

